# Outcomes of Conservative Versus Surgical Treatment of Dropped Head Syndrome in a Single Institution: A Case Series and Review of the Literature

**DOI:** 10.7759/cureus.80777

**Published:** 2025-03-18

**Authors:** Serena B Liu, Catherine E Wassef, Addisu Mesfin, Robert Molinari

**Affiliations:** 1 Orthopedic Surgery, University of Rochester Medical Center, Rochester, USA; 2 Neurosurgery, University of Rochester Medical Center, Rochester, USA

**Keywords:** camptocephalia, cervical deformity, cervical kyphosis, chin-on-chest deformity, dropped head syndrome

## Abstract

Dropped head syndrome (DHS), also known as camptocephalia, is characterized by a mobile chin-on-chest deformity from hypotonia of the cervical extensor muscle or hypertonia of the anterior neck muscles. There remains a paucity of quality published literature on this topic, particularly its management. The objective of this study is to identify radiographic and clinical outcomes of surgery as compared to nonsurgical treatment, to summarize the literature, and to create a decision-making paradigm for managing patients with DHS. As such, we report outcomes of our retrospective chart review series as well as a literature review on the etiology, management options, and outcomes. In our series, we examined the radiographic parameters of the C2-C7 Cobb angle, C2-C7 sagittal vertical axis (SVA), and T1 slope, as well as clinically reported outcomes of conservatively managed (CM; N = 8) and surgically managed (SM; N = 5) interventions on 13 patients with DHS at a single institution. At initial presentation, the CM group had poorer C2-C7 Cobb angle (-37.8 ± 3.2 degrees) and C2-C7 SVA (77.1 ± 10.6 mm) in comparison to the SM group (-21.0 ± 30.3 degrees and 56.9 ± 16.2 mm, respectively). We found a statistically significant improvement in the C2-C7 Cobb angle and T1 slope (p-value of 0.024 and 0.019, respectively) after surgery. Clinically, only one patient in the CM group (20%) reported improvement versus six patients in the SM group (80%). Our study is limited by its small sample size, albeit it is the largest cohort of patients treated at a single institution in the United States to our knowledge. Although our cohort was comprised of heterogeneous etiologies and patient comorbidities, we found that surgery can be beneficial in the right patient. To aid in proper surgical selection, we provide an algorithm for workup and management of DHS.

## Introduction

Dropped head syndrome (DHS), also known as camptocephalia, is a debilitating clinical condition in which a person cannot hold his/her head upright due to an imbalance in cervical neck extensor and flexor muscles. It is a mobile chin-on-chest deformity and interferes with the patient’s daily function. The incidence is unknown, but one meta-analysis found the mean age is 63.6 years and 63% of the patients were female [1].

There is a myriad of etiologies that have been published over the years since it was first identified. It can also be classified as neuromuscular etiology and non-neuromuscular etiology. Of the neuromuscular etiologies, many diseases have been identified, such as Parkinson’s disease, post-polio syndrome, amyotrophic lateral sclerosis (ALS), and myasthenia gravis [1]. There are several different etiologies for non-neuromuscular causes, such as post-radiation exposure, post-traumatic changes, and idiopathic.

Other case reports and small case series have been published. However, there remains a paucity of quality published literature on this topic. In this report, we analyzed the largest case series in the United States to date with a case series of 13 patients who presented to a single academic medical center during a 10-year period. Our primary objective is to report the management of our patient series and review the current literature. Our secondary objective is to examine outcomes between surgical and conservative management after stratifying by etiology. We hypothesize that patients who received surgical intervention had improved outcomes in comparison to conservative management of DHS.

## Materials and methods

After obtaining approval from the institutional review board, a retrospective chart and imaging review was conducted for all identified patients diagnosed with DHS by one of the three fellowship-trained orthopedic surgery spine surgeons at a single institution between April 2012 and February 2022.

Chart review

We collected demographic data, comorbidities, etiologies, clinical history, physical exam, duration of follow-up with the orthopedic surgeon, and types of treatment received. For the surgical group, we also examined levels of fusion and perioperative complications. Clinical vignettes are provided in the Appendices. All patients were offered conservative management prior to surgical treatment.

Radiographic parameters

**Figure 1:**
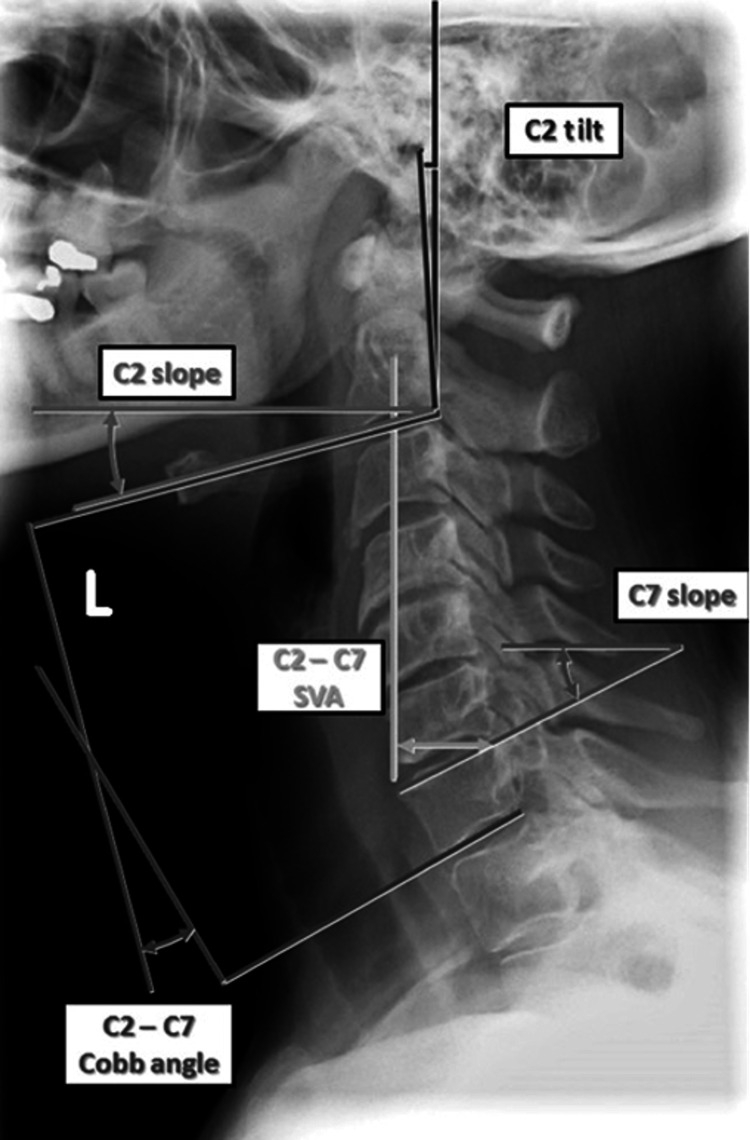
Pictorial representation of measurements calculated on lateral cervical spine radiographic imaging. C2-C7 cobb angle is obtained by drawing a line parallel to the inferior endplate of C2 and the inferior endplate of C7. C2-C7 SVA is the horizontal offset from a plumb line originating at the C2 vertebral body center to the posterosuperior corner of the C7 vertebral body. T1 slope is the angle between the upper-end plate of T1 to a horizontal line. SVA: sagittal vertical axis.

Furthermore, we analyzed the C2-C7 Cobb angle, C2-C7 sagittal vertical axis (SVA), and T1 slope on upright cervical spine imaging at initial clinic visits (Figure 1). For the surgically managed group, the above radiographic measurements were repeated on postoperative imaging obtained six to 12 months after surgery.

Statistical analysis

Statistical analysis was performed with Microsoft® Excel Data Analysis version 16.60 (Microsoft Corporation, Redmond, WA). Descriptive statistics (median and interquartile range) were calculated for all variables.

Paired t-tests were used to compare baseline demographic and radiographic parameters for both operative and nonoperative patients. For the operative patients, Wilcoxon signed-rank tests were performed between the pre- and postoperative C2-C7 Cobb angle, pre- and postoperative C2-C7 SVA, and pre- and postoperative T1 slope.

## Results

Case series

We had a total of 13 patients in our study. Five patients in the conservatively managed (CM) group (cases 1-5) and eight patients (cases 6-13) in the surgically managed (SM) group (Table 1). The average age in the conservative group (74 years old) was older than the SM group (68 years old). There were three females in the CM group (60%), and two females in the SM group (25%). All patients demonstrated chin-on-chest deformity on physical exam. Presentation ranged from acute onset within weeks to gradual onset over the span of years in both groups. The median duration of follow-up in the CM group was 0.0 weeks (IQR: 0.0 - 13.8), which was shorter than the SM group at 83.8 weeks (IQR: 70.6 - 122.4). In the SM group, most of the constructs were from C2 (75%) to the upper thoracic spine, with a median of 11 levels (IQR: 8 - 11) included in the fusion construct. A detailed description of each patient is included in the Appendices.

**Table 1:** Clinical summary of the patients. ALS: amyotrophic lateral sclerosis; PSIF: posterior spinal instrumented fusion.

Case	Age	Sex	Features	Comorbidity	Onset	Etiologies					Length follow-up (weeks)		Improvement		
Non-operative management															
1	81	M	Chin-on-chest deformity. Passively correctable.	ALS	Gradual - 1 year	ALS					0.0		Yes		
2	73	F	Chin-on-chest deformity. Passively correctable.	Osteoporosis	Acute - 3 weeks	Idiopathic					0.0		No		
3	71	F	Chin-on-chest deformity. Occurred after C4-5 PSIF for unstable C4-5 posterior element fracture. Passively correctable.	Hypothyroidism	Acute - 6 weeks	Post-surgical (3/31/2014)					75.0		No		
4	65	M	Chin-on-chest deformity. Passively correctable.	Hodgkin's lymphoma with extensive radiation treatment	Gradual - unknown	Post-radiation					13.6		No		
5	80	F	Chin-on-chest deformity. Passively correctable.	Non-Hodgkin's lymphoma with extensive radiation treatment	Gradual - 10 years	Post-radiation					0.0		No		
Mean	74								Median (IQR)		0.0 (0.0-13.8)				
Operative management															
							Levels of fusion (number of fusion levels)		Length from diagnosis to surgery (weeks)						
6	74	M	Chin-on-chest deformity. Unable to extend the neck to neutral.	Diabetes mellitus, Parkinson’s disease, early dementia	Gradual - 18 months	Idiopathic		C2-T6 PSIF (12)		19.6	76.6		Yes		
7	80	M	Chin-on-chest deformity. Passively correctable.	Diabetes mellitus, osteoporosis, and emphysema	Acute - 4 weeks	Idiopathic		C2-T3 PSIF (9)		6.1	18.0		Yes		
8	56	M	Chin-on-chest deformity. Passively correctable.	Diabetes mellitus, extensive radiation therapy for Hodgkin's lymphoma	Gradual - unknown	Post-radiation		C2-T5 PSIF (11)		55.0	177.4		No		
9	78	F	Chin-on-chest deformity. Occurred after C1-C2 posterior fusion surgery for odontoid fracture. Passively correctable.	Osteoporosis	Acute - 11 days	Traumatic		Halo		0.3	104.0		Yes		
10	64	M	Chin-on-chest deformity. Occurred after C4-C5 compression fractures. Passively correctable.	Parkinson’s disease, mantle cell lymphoma, metastatic colon cancer	Acute - 1 month	Traumatic		C2-T1 PSIF (7)		0.9	83.3		No		
11	63	M	Chin-on-chest deformity. Unable to passively correct past neutral.	Prostate cancer	Gradual - unknown	Post-surgical		C2-T1 PSIF (7)		2.9	52.7		Yes		
12	69	M	Chin-on-chest deformity. Occurred after C1-2 posterior fusion. Passively correctable.	Diabetes mellitus	Gradual - 2 years	Post-surgical		O-T3 PSIF (11)		55.1	221.6		Yes		
13	63	F	Chin-on-chest deformity. Occurred after C3-C7 laminectomy, C2-T1 PSIF for cervical degenerative spinal stenosis.	Rheumatoid arthritis, post-polio syndrome	Gradual - 4 months	Post-surgical		C2-T5 PSIF (11)		16.1	84.3		Yes		
Mean	68					Median (IQR)		11 (8-11)		11.1 (2.4-28.4)	83.8 (70.6-122.4)				

Clinical outcomes

In the CM group, only one out of the five patients (20%) reported clinical improvement with conservative management such as physical therapy and collar.

In the SM group, six of the eight patients reported clinical improvement (80%). The remaining two patients who did not report clinical improvement had progressive kyphosis with associated dysphagia upon neck extension. Two patients (20%) suffered complications. One patient had superficial wound dehiscence and subsequent infection at one year postoperation requiring operative irrigation. Another patient experienced C2 ramus occipital neuralgia after halo reduction of the fracture, and no posterior instrumentation was utilized.

Radiographic parameters

Only one patient in the CM group had a second set of radiographic imaging; hence only initial upright cervical radiographic images were analyzed for the CM group (Table 2). For the CM group, the median C2-C7 Cobb angle, C2-C7 SVA, and T1 slope angle were -37.7 degrees (IQR: -39.1 to -36.7), 74.7 mm (IQR: 70.3 - 76.9), and 33 degrees (IQR: 22.5 - 37.6), respectively, on upright cervical spine radiographic imaging.

**Table 2:** Radiographic parameters for nonoperative, preoperative, and postoperative images. SVA: sagittal vertical axis.

		C2-C7 Cobb angle (degree)			C2-C7 SVA (mm)			T1 slope angle (degree)		
Nonoperative										
1		-39.1			68.6			33		
2		-36.7			76.9			57		
3		-42.1			74.7			22		
4		-37.7			95.1			37.6		
5		-33.3			70.3			22.5		
Median (IQR)		-37.7 (-39.1 to -36.7)			74.7 (70.3 - 76.9)			33 (22.5 - 37.6)		
Operative										
		Pre-op	Post-op		Pre-op	Post-op		Pre-op	Post-op	
6		-50	1		75.9	31.6		31.8	18.5	
7		-25.1	25.5		75.5	52.3		48.3	68.8	
8		24.9	4.2		31.5	73.8		28.3	43.2	
9		-1	-5.4		63.2	63.7		38.3	54.6	
10		-15.7	7.1		62.3	70.8		29.1	60.3	
11		-58	2.2		37	25.1		0	26.4	
12		-50.6	4.7		51.4	74.4		3.5	65.5	
13		7.3	6.7		58.1	34		26.5	27.8	
Median (IQR)		20.4 (50.2 to -1.1)	4.5 (1.9 - 6.8)		60.2 (47.8 - 66.3)	58 (33.4 - 71.6)		28.7 (20.8 - 33.4)	48.9 (27.4 - 61.6)	

At initial presentation, the SM group had median C2-C7 Cobb angle, C2-C7 SVA, and T1 slope angle of 20.4 degrees (IQR: -50.2 to -1.1), 60.2 mm (IQR: 47.8 - 66.3), and 28.7 degrees (IQR: 20.8 - 33.4) respectively. The mean postoperative C2-C7 Cobb angle, C2-C7 SVA, and T1 slope angle were 4.5 degrees (IQR: 1.9 - 6.8), 58 mm (IQR: 33.4 - 71.6), and 48.9 degrees (IQR: 27.4 - 61.6), respectively. There was statistical significance with a two-tailed Wilcoxon signed rank test for the T1 slope angle with a Wilcoxon signed rank statistic of 2 (within the critical value of 3 for a two-tailed test with eight entries at ⍺ = 0.05). There was no statistical significance with C2-C7 Cobb angles or SVA (Wilcoxon signed rank statistic was 6 and 14, respectively). P-values were unable to be determined given the size of the population.

## Discussion

Review of the literature

Definition of Dropped Head Syndrome

DHS does not have a radiographic or calculated definition, but a clinical diagnosis of mobile chin-on-chest deformity. In assessing for improvement in patient outcomes, we cannot ignore the changes in cervical and overall spine alignment. There are several cervical spine alignment parameters used when assessing DHS. In our review, we have found four types of measurements that correlate with age and some with quality of life. Firstly, the chin-brow vertical angle, a common measure of horizontal gaze, requires radiographs to include the chin and brow in a standing film [2]. Although an ideal measurement for DHS, we were unable to acquire this information due to the retrospective nature of our data collection.

Another measurement, the C2-C7 Cobb angle (or lordosis) has been found to be positively correlated with age [3]. A higher Cobb angle indicates more cervical kyphosis. A study of 1200 asymptomatic volunteers found the average lordosis in the C2-C7 region was 13.9 + 12.3 degrees [4]. In conjunction with the T1 slope, this parameter has been implicated in health-related quality-of-life outcomes [5]. When the T1 slope and C2-C7 lordosis mismatch is greater, the patients rated greater disability on the Neck Disability Index (NDI) [5]. Another measurement, the C2 slope, has also been correlated with the T1 slope minus C2-C7 lordosis, in addition to postoperative pain on disability scores [6]. Given the retrospective nature of our study, the C2 slope and C2-C7 Cobb angle were the most universally acquired data points for our calculations.

As in the lumbar spine, a measure of sagittal balance can be obtained by using the C2-C7 SVA by measuring the horizontal distance between the plumb lines drawn down from the superior posterior corner of the C7 vertebral body and the center of the C2 vertebral body [2]. In standing, asymptomatic volunteers, the C2-C7 SVA was 16.8 + 11.2 mm [2]. Miura et al. [7] showed that this is a dynamic value, with a gradual increase in cervical SVA from 75 to 85 mm during ambulation in one patient complaining of neck pain with ambulation. Thus, calculations obtained on stationary radiographs may underestimate the amount of strain placed on the neck during ambulation in decompensated cervical kyphosis. A recent study has divided DHS into positive and negative SVA while analyzing the thoracolumbar compensation mechanisms for each [8]. According to the authors, identifying positive and negative SVA types has implications for surgical construct levels [8]. Other studies have correlated cervical SVA with health-related quality-of-life outcome measures [9,10]. For the purposes of this study and many others, success/improvement is a subjective measure reported by the patient during clinic visits [11,12].

Etiology of DHS

There are three main categories for the etiology of DHS: medical/systemic, radiation-induced, or post-surgical/trauma-induced. The medical etiologies can be further dissected into neurological, neuromuscular, muscular, and systemic inflammatory diseases [13]. Please refer to Table 3 for the comprehensive list.

**Table 3:** Etiologies of DHS. DHS: dropped head syndrome.

Etiology	Examples in literature
Neurological	Amyotrophic lateral sclerosis (ALS), Parkinson's disease, multiple system atrophy, cervical dystonia, post-polio syndrome, cervical myelopathy, syringomyelia, chronic inflammatory polyneuropathy (CIDP), hemispheric striatal infarction, frontotemporal lobar degeneration with ubiquitin inclusions.
Neuromuscular	Myasthenia gravis (MG), Lambert-Eaton myasthenia syndrome (LEMS).
Muscular	Isolated neck extensor myopathy (INEM), primary inflammatory such as polymyositis, scleromyositis, isolated inflammatory axial myopathy, anti-GAD-associated inflammatory myopathy, primary non-inflammatory such as nemaline myopathy, myopathy with rimmed vacuoles, myofibrillar myopathy, necrotizing autoimmune myopathy, inclusion body myositis, mitochondrial myopathy, muscle creatinine deficiency, congenital muscle dystrophy, facioscapulohumeral muscular dystrophy (FSHD), primary amyloidosis, muscle-restricted vasculitis, sarcopenia, cancers like paraspinal neuroblastoma.
Systemic	Systemic sclerosis, scleroderma-associated myopathy, drug reactions, hyperparathyroidism, hypothyroidism, hypokalemia, B-cell chronic lymphocytic leukemia-myopathy.
Trauma and iatrogenic	Spinal cord injury, serial botulinum toxin injections, multilevel cervical radiofrequency ablation, and post-surgical dropped head syndrome.
Post radiation	Hodgkin's lymphoma, mantle irradiation therapy, nasopharyngeal cancers.

Trauma and iatrogenic examples include spinal cord injury [14], serial botulinum toxin injections [15], multilevel cervical radiofrequency ablation [16], and post-surgical DHS. One study found that preoperative flexion/extension range of motion correlates with post-laminoplasty kyphosis [17]. If the preoperative difference in range of motion (measured as C2-C7 Cobb angle in flexion vs. neutral and extension vs. neutral) between flexion and extension is greater than 30 degrees, patients were more likely to have a loss of cervical lordosis. A higher degree of flexion is also associated with a higher likelihood of cervical kyphosis (40.2 ± 8.8 for patients who developed kyphosis postoperatively vs. 26.6 ± 9.6 for patients who did not develop kyphosis). Thus, preoperative flexion and extension films should be taken into consideration before cervical laminectomy or laminoplasty procedures.

The pathophysiology behind post-radiation is not fully understood. It seems that some cancer types are more likely than others to get DHS, with one study quoting about 83% of Hodgkin’s lymphoma (HL) patients getting DHS post-radiation [18]. The most reported cancer associated with post-radiation DHS is HL, although some nasopharyngeal cancers have also been associated with DHS [18-21]. One study looking specifically at post-HL radiation DHS patients had a heterogeneous mix of electromyography (EMG) and pathology findings, with some neurologic pathology and some muscular pathology [22]. The onset of DHS from completion of radiation in one study was between five and 15 months (n = 3) [23]. Radiation dosage to neck extensor muscles is a likely factor, where the majority of DHS radiated patients in one study had >46 Gy to the extensor muscles. The authors conclude by proposing <46-50 Gy of radiation as the limit of radiation to neck extensor muscles [23].

Clinical Investigation of DHS

The workup of DHS should be multimodal and help diagnose etiologies that tend to resolve spontaneously or with medical management. The pillars of diagnosis are the history, physical exam, electrodiagnostic studies, imaging, blood work, and muscle biopsy. On physical exam, the kyphosis should be mobile to qualify for DHS. Cervical extensor myopathy may be visualized as atrophy of the cervical extensors, which points to a neuromuscular disorder [24]. In non-neuromuscular causes, dystonic anterocollis may be observed [24]. Bulging of the cervical muscles may also be observed [25]. A study of 107 patients found that reports of limb weakness and neck flexor weakness were associated with medical treatment responsiveness [19].

Spinal imaging is generally performed to rule out structural diseases of the cervical spine, such as fractures/ligamentous instability. Cervical spine MRI is the imaging modality of choice given its imaging quality, the ability to see neural elements, as well as cervical musculature. Inflammatory isolated neck extensor myopathy (INEM) can best be seen on T2-weighted (+ fat suppression), turbo spin echo (TSE), or short tau inversion recovery (STIR) images as increased signal intensity in the muscle [26]. Given its response to medical treatment, INEM should be sought out when looking at the MRI [26]. In addition to looking for etiologies of the DHS on imaging, overall spinal balance parameters can be useful in determining the success of treatment, whether it be medical, conservative, or surgical. For this reason, scoliosis standing plain films should be obtained to assess for SVA, pelvic tilt, lumbar lordosis, and other spinal alignment measurements [11]. Kusakabe et al. [11] found that the patients with normal SVA (between -30 and 40 mm) with acute non-traumatic DHS were most likely to recover with conservative treatment.

Given the fair number of metabolic diseases that could produce DHS, a blood panel should be ordered to look for inflammatory markers or markers of infection. Specifically, serum creatine kinase, serum monoclonal gammopathy, acetylcholine receptor antibody (myasthenia gravis), blood-cell count, basic metabolic panel, C-reactive protein, serum protein immunoelectrophoresis, thyroid function test, parathyroid hormone level, and serologies for human immunodeficiency virus 1 and 2, and B and C hepatitis virus [19,26,27].

EMG is a useful tool to assess for muscular disease or nerve-mediated disease. A study of 16 patients reporting DHS found tonic EMG activity in the neck flexors when patients were supine, with some tonic activity in the neck extensors when sitting or standing [25]. For focal myositis of the neck extensor muscles, and more broadly, isolated neck extensor myopathy, the pathology is localized to the neck extensor muscles, so it is of utmost importance to test the cervical paraspinal muscles specifically, although some studies have also found abnormalities in the upper and mid-thoracic paraspinal muscles [28]. Several studies have shown that cervical paraspinal muscles can exhibit fibrillations, positive sharp waves, and many motor units of <2-millisecond duration with early recruitment [26,28]. In one EMG study of post-Hodgkin's lymphoma radiation-associated DHS, results were heterogeneous for nerve-mediated disease vs. myopathy [22]. Some patients had neurogenic EMG changes in the splenius capitis, upper trapezius, supraspinatus, and infraspinatus, one had reduced compound muscle action potential amplitude in the upper trapezius with spinal nerve stimulation, while three others had abnormal muscle activity (fibrillation potentials or complex repetitive discharges) [22]. Identifying myopathy or nerve-mediated disease can help determine treatment options.

Management of DHS

Muscle biopsy is a useful tool to diagnose several myopathies. According to a study of 107 patients, splenius capitis had the most diagnostic yield, with 67% of biopsies from this area leading to a diagnosis as opposed to limb muscle biopsies, which had a 42% diagnostic yield [19]. Idiopathic restricted non-inflammatory myopathy, which is a type of isolated neck extensor myopathy, shows a necrotizing myopathy without inflammation on biopsy [29]. Other types of isolated neck extensor myopathy show only fibrosis without signs of myositis [30]. In focal myositis of the neck extensor muscles, biopsy shows necrosis of muscle fibers, myophagia, and T cell infiltrates [26]. Myositis can also include massive inflammatory infiltrates of B and T-cells, plasma cells, and macrophages in both the perivascular and endomysial compartments [31].

The management of this syndrome is as heterogeneous as the etiologies are. There are four broad categories of treatment: medical, rehabilitation, orthotics, and surgical. It appears that for certain etiologies, medical management and rehabilitation are acceptable options. Surgical management overall seems to have a favorable result, based on the review of the literature. We will go through each option in detail.

Medical management is an option for patients who have a medical etiology for their issues. Patients with underlying INEM without myositis, polymyositis, myositis, or other inflammatory processes should get steroids, with several authors describing a prednisone regimen lasting two to four months [19,26,30-33]. Alhammad and Naddaf [19] did a retrospective review of DHS patients and found them to all have some form of myopathy, with 53% responding to steroids, immunosuppressants, or a combination of the two. Subcutaneous immunoglobulin therapy has been described as effective in a case report of myopathic neck extensor weakness secondary to systemic sclerosis and subsequent common variable immunodeficiency [34]. Underlying systemic medical diseases, such as myasthenia gravis (MG), should be treated with the therapy of choice for that disease (i.e., pyridostigmine for MG and parathyroidectomy for primary hyperparathyroidism) [27,28]. There have also been reports of spontaneous resolution of DHS due to INEM four months after presentation [35].

Rehabilitation programs have been described in varying levels of detail as a first-line conservative management option before surgery. The effectiveness of physical therapy is high in some studies (63% effective) while low in others [19]. In studies pointing to the significant effectiveness of physiotherapy, the regimen tends to be time-intensive. An outpatient physical therapy regimen should include posture guidance and strengthening of cervicothoracic extensor muscles as well as balance [11]. Other descriptors of intensive rehabilitation involve 40 months of ﻿cervical and lumbar manipulation, spinal traction, micro-vibration deep muscle massage, and core muscle training with resolution of DHS [36]. A small series of 18 patients underwent an intensive surgical inpatient rehabilitation program for two weeks and saw improvements in the chin-brow vertical angle and the NDI score [37]. However, a meta-analysis done in 2019 of 129 patients revealed no benefit to physical therapy/orthotics; in fact, physical therapy trended toward becoming a predictor of a negative outcome [1]. One way to reconcile these two findings is that the type of physical therapy is most relevant to what outcome ensues. Another explanation may be that certain etiologies, like trauma, do not improve with physical therapy while acute non-traumatic DHS may improve with an intensive physical therapy regimen [11].

Aside from the standard orthotics of halo or cervical collar, there has been one orthotic described specifically for DHS. The “baseball cap” orthotic has four components. A strap is applied to the back of a baseball cap and a thoracic rod is attached to it [38]. The rod is then fixed to the torso with another circumferential strap. Fast et al. [38] published a two-patient series on its use; one with post-laminectomy syndrome (post-polio syndrome with C5-6 laminectomy) who progressed to fusion and the other with multiple myeloma (EMG confirmed myopathy that did not respond to steroid treatment) who found it to be intermittently helpful. There is no evidence that this orthotic has treated DHS successfully in any objective manner but could potentially be an adjunct to physical therapy. There have been case reports of intensive rehabilitation with a portion in cervical traction and halo for eight weeks and a total of 10 weeks of treatment [39]. The patient was then able to lift her head after treatment and results were sustained at a two-year follow-up [39].

If a surgical option is considered, a multidisciplinary approach to the patient’s preoperative and postoperative course must be implemented. Given many of these patients have several co-morbidities, a preoperative workup should be thorough. Given the mechanical disadvantage associated with DHS, swallowing can be very difficult and up to 50% of DHS patients will have abnormalities on swallow evaluation [19]. Pulmonary function may also be affected due to partial obstruction of the trachea or pharynx, with 89% of patients having abnormal pulmonary function tests in one study [19]. Alhammad et al. [19] also found that 37% of their DHS patients had an abnormal electrocardiogram and 13% had an abnormal echocardiogram. Because of the involvement of all these organ systems, preoperative counseling should include a discussion of possible temporary tube feeding and consultation with the anesthesiology team. Communication between the anesthetic and surgical teams prior to surgery is paramount for optimal surgical outcomes.

There are several considerations when thinking about surgically approaching DHS. The etiology of the DHS, neurologic status, and what surgical approach to take are all factors to be considered. Regarding whom would most benefit from surgery, it appears in the literature that DHS secondary to trauma responds favorably to surgical intervention, as one would expect [40,41]. Neurological deficits and symptoms of myelopathy are strong indications for surgery if conservative management has failed [42]. In our series, we did not see a difference in improvement between surgical patients with acute or gradual onset of DHS, although previous studies have reported better resolution of DHS for acute onset patients, regardless of surgical intervention [11].

Given the deformity nature of this issue, nearly all patients require some sort of instrumentation and fusion. In our series, one patient’s operative intervention was only a halo placement, but this patient suffered from post-surgical DHS and already had hardware in (C1-2 fusion). The halo was used to help maintain alignment until the fusion partially healed and the tissues were scarred in place. In most cases reported in the literature, constructs extend posteriorly from C2 to the high or middle thoracic spine [12,41-44]. Kudo et al. [45] found in a study of 41 case-matched DHS patients that cervical spine imaging of DHS patients showed lower-level dominant severe degenerative change and upper-level dominant spondylolisthesis, which might explain why constructs generally span the entire cervical spine and anchor into the thoracic spine. A recent systematic review of surgical management of DHS indicated a 71% failure rate for fusions terminating above the thoracic spine, as opposed to a 13% failure rate for cervicothoracic constructs [46]. A combined anterior/posterior approach seems to have better radiographic results, specifically restoration of lordosis, but higher rates of dysphagia (75%) [46]. According to Qian et al. [8], patients with positive cervical SVA should have constructs terminating in the thoracolumbar spine due to lack of thoracolumbar compensation, but that has not been our experience with positive cervical SVA.

Regarding the need for anterior column support or release, one study seemed to indicate that radiation-induced DHS is more prone to requiring anterior column release [12]. A study by Smith et al. polling 14 deformity surgeons on 18 cervical deformity cases, with two “chin on chest” cases, found that for chin-on-chest deformity, 82% of surgeons would perform posterior surgery only, with 15% of surgeons performing both anterior and posterior surgery, and 3% performing back-front-back or front-back-front surgery [47]. The average number of anterior levels was 4.5 and the posterior was 13.2. Ultimately, surgical management should be considered on a case-by-case basis.

Discussion of case series

We have presented a case series of 13 patients with DHS. The etiologies were diverse; three patients had idiopathic onset, four were post-surgical, two were post-traumatic, three were post-radiation, and one was due to amyotrophic lateral sclerosis. We will discuss each etiology and management, followed by outcomes and a proposed algorithm.

For patients who have received head and neck radiation, DHS is a known latent complication [21]. Inaba et al. noted that a radiation dose of >50 Gy for head and neck carcinoma to the cervical extensor muscles increases the risk of DHS, as it was present in two of the three patients in their study [23]. In our series, all post-radiation patients had a history of lymphoma, and one out of three underwent surgical intervention. This patient received a C2-T5 fusion with an iliac crest bone graft but unfortunately had several complications, including pressure ulcers at the wound and proximal junction kyphosis. Based on this experience, it is unclear if a longer construct is necessary to prevent proximal junctional kyphosis (PJK) in the setting of compromised musculature secondary to radiation. Our literature review pointed to patients with radiated DHS requiring anterior column release, which may have been the answer in this patient [12]. The other two have not undergone surgery, although one had established failure of conservative management.

Our series included four post-surgical DHS patients, three of whom had undergone posterior fusions (C1-2, C2-T1, C4-5) and one had a laminoplasty (C3-6). Three of the four patients underwent surgery, specifically fusion extension, and saw improvement in their symptoms. Unfortunately, the patient with a previous C4-5 fusion did not improve with conservative management.

Regarding idiopathic etiologies, one theory is that weakness or laxity of the semispinalis cervical muscle causes progressive kyphosis and degeneration to the point of DHS [48]. If the semispinalis cervical muscle is intact, strengthening this muscle could reverse DHS. This could also explain post-surgical kyphosis in posterior approaches, disrupting this muscle and compromising the posterior musculature. Regarding treatment for the idiopathic type, we treated two out of the three patients with surgical intervention, and these patients showed post-intervention improvement. The one patient who was followed conservatively reported continued pain but did not want surgery and ultimately died from unrelated causes. We did not test our idiopathic patients for underlying autoimmune or muscular disorders. This, in addition to muscle biopsy, is likely to be beneficial in the workup of idiopathic DHS in the future.

Cervical spine traumatic injuries are variable, and our series includes two patients with cervical spine injuries; one with a C1 and C2 fracture/nonunion and the other with a C4-5 fracture. The high cervical patient underwent traction, realignment, and halo placement, whereas the patient with C4-5 fracture underwent an anterior corpectomy and posterior fusion. Both patients complained of significant and continuous neuropathic pain beyond the perioperative period. However, imaging for both showed good fusion without abnormal mobility. This highlights the importance of a multidisciplinary team that can assist with pain management after radiographic success has been achieved.

Finally, ALS is a known neuromuscular cause of DHS, with DHS occurring in 1-3% of ALS patients [49]. One study noted that patients with proximal leg weakness did not develop DHS, but patients with DHS initially presented with bulb palsy and upper limb weakness. Another study of 683 ALS patients found that DHS occurs early on the course, approximately one to two years from diagnosis. The leading theory is a progression of extensor muscle weakness that exceeds flexor muscle weakness. Our case series included one patient with ALS, who was ultimately treated conservatively with a Miami J collar, physical therapy, medications, and radiofrequency ablation of medial cervical branches.

**Figure 2:**
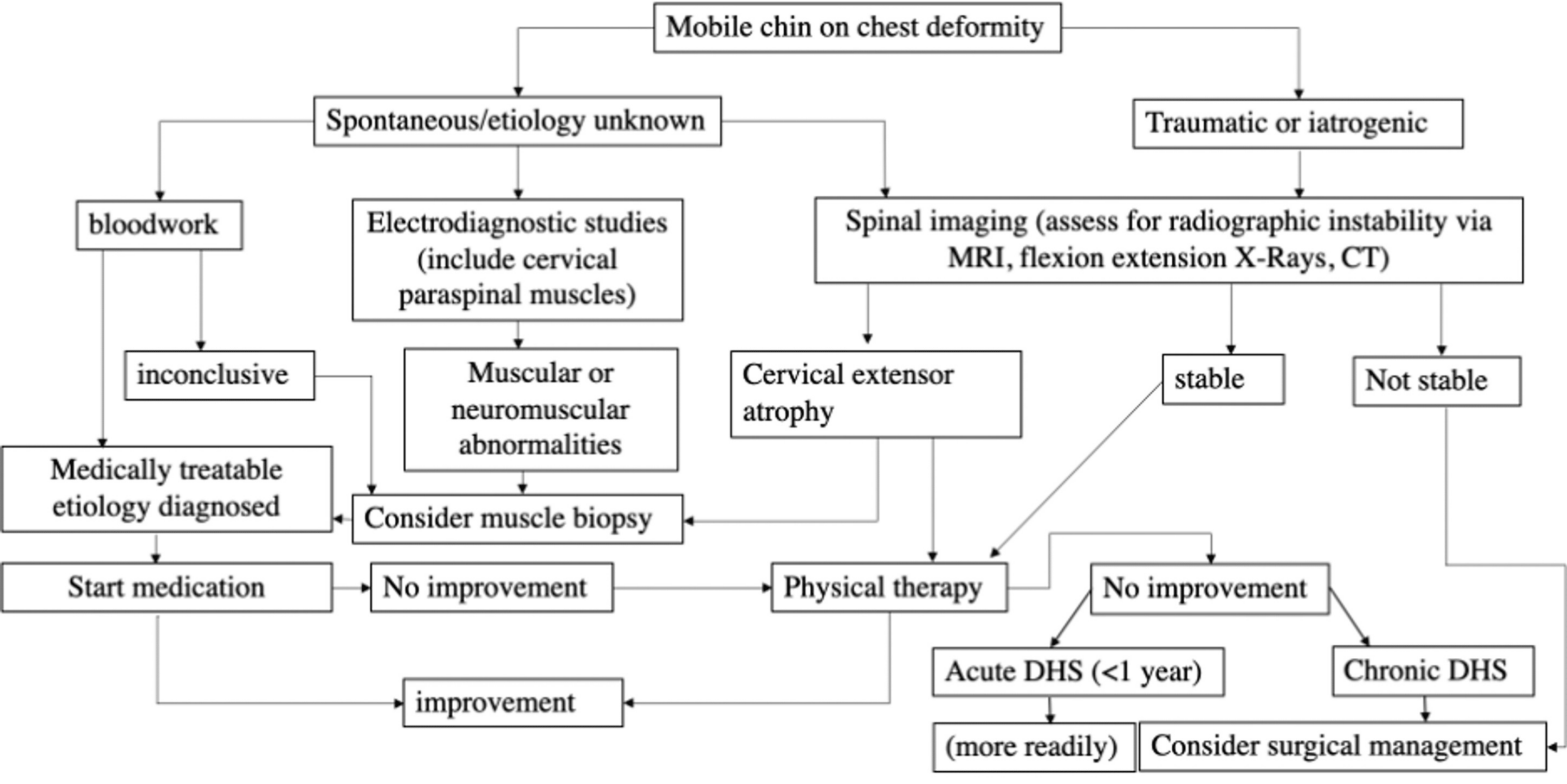
Proposed algorithm for workup and management of DHS. DHS: dropped head syndrome.

An algorithm has been proposed in the past regarding the management of the DHS (Figure 2). The first step is to establish mobility, distinguishing DHS from chin-on-chest deformity and other fixed pathologies. The next step is to work up for neuromuscular disorder and manage the condition as appropriate. If there is no neuromuscular disorder, treat conservatively with physical therapy and a cervical collar. If there is no improvement, then surgery can be considered. This allows for a trial of strengthening of neck extensor muscles and potential avoidance of surgical intervention, which can have its own set of complications.

Based on our analysis, surgery was able to improve the Cobb angle and T1 slope, and patients reported improvement in pain. These data are consistent with the literature descriptions of C2-C7 and T1 mismatch causing neck pain. Given the heterogeneous etiologies of our patient’s DHS, we acknowledge that not all patients will benefit from surgery. However, it seems that in our series, post-surgical patients benefitted from surgery. We did not see any advantage to physical therapy in the conservatively managed group, but this may be due to the heterogeneous administration of “physical therapy” and decreased follow-up in this cohort.

Limitations and future directions

We acknowledge that our conclusions are limited by the descriptive nature and small volume of the study as a case series. Given the heterogeneity of our DHS population and their disease management as well as limited long-term follow-up, the need for a case-control or randomized control trial is evident but not likely given the rarity of the disease. A prospective longitudinal series would be helpful to obtain long-term outcomes in the future.

## Conclusions

Surgery is a viable option for deformity correction of DHS, although surgical candidates should be selected wisely. Patients should have failed conservative management before consideration for surgery. Patients with acute onset DHS and a history of trauma/prior surgery tend to have a better clinical outcome and better deformity correction. We propose an algorithm be used from presentation to treatment for patients with DHS.

## Figures and Tables

**Figure 3:**
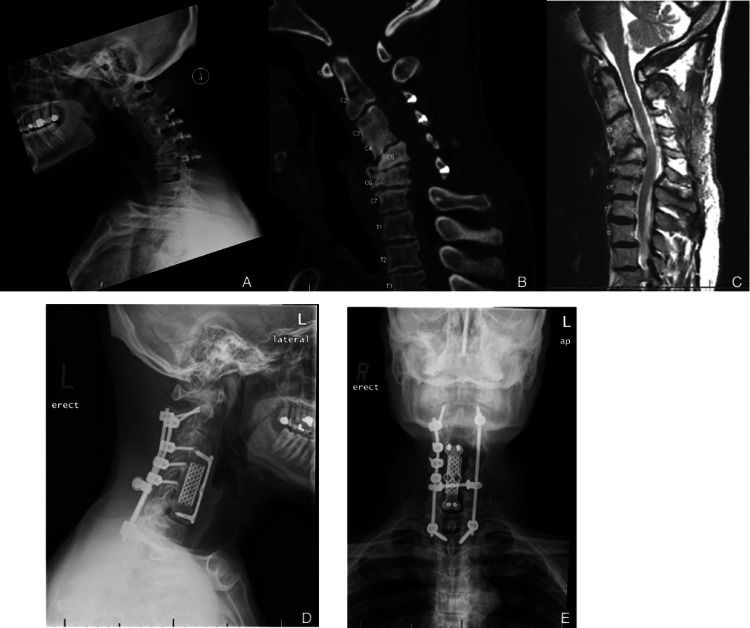
Pre- and postoperative images of a post-cervical laminoplasty cervical kyphosis patient. The patient presented with loss of neck mobility and difficulty swallowing. Note the significant swan neck deformity (Panel A) and C3-4 autofusion (Panel B) without cord compression (Panel C). The patient opted for surgical correction. Anteriorly, the patient was placed in Gardner Wells tongs, followed by C5 and C6 corpectomy, C4-5 and C6-7 discectomy, C5-6 cage, and C4-7 plate. The patient was then placed in the Mayfield, flipped prone, and underwent C2-T1 fusion and C3-7 decompression. Note the correction of the swan neck deformity and restoration of sagittal balance in the lateral radiograph (Panel D) and elevated chin in the anteroposterior radiograph (Panel E). The patient remains well eight years postoperatively.

**Figure 4:**
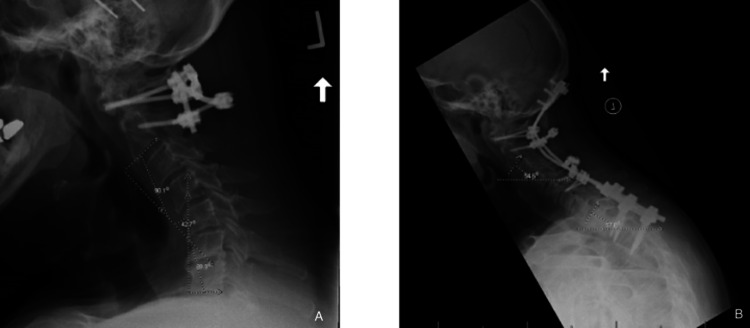
Pre- and postoperative images of a patient with post-surgical dropped head syndrome (DHS). A 79-year-old male presented five months after C1-2 fusion for type II odontoid fracture and Jefferson fracture (Panel A) with mobile chin-on-chest deformity. Eight months after the original surgery, the patient underwent the removal of hardware, followed by occipital to T3 posterior instrumented fusion (Panel B). At six months post operation, the patient had improvement in his symptoms and acceptable clinical kyphosis. At a three-year follow-up, the patient complained of shuffling gate and axial neck pain. He was referred to physical therapy for balance training and muscle strengthening.

## Appendices

Clinical vignettes

Case 1: Amyotrophic Lateral Sclerosis (ALS)

The patient was an 81-year-old male who presented with an insidious onset of head ptosis that worsened over one year. It was associated with imbalance during walking, and radicular pain radiating from the neck into the right shoulder and arm. His wife also noted a change in his voice and enunciation, which she attributed to the position of this head. Physical exam demonstrated passively correctable chin-on-chest deformity with the inability to actively maintain the head in a neutral cervical stance or extension. The exam was otherwise unremarkable aside from a positive Romberg sign. Radiographic cervical imaging showed multilevel degenerative disc disease with a kyphotic alignment. The patient was prescribed physical therapy for gait training and a Miami J collar. He was also referred to neurology, where the patient was diagnosed with ALS after EMG showed widespread denervation and reinnervation.

The patient decided to pursue conservative treatment consisting of Miami J collar, physical therapy, medication, and radiofrequency ablation of medial cervical branches until he deceased from undocumented reasons. DHS failed to improve with nonoperative measures.

Case 2: Idiopathic DHS

A 72-year-old female with a past medical history of osteoporosis presented with a three-week history of insidious axial neck pain with radiation into the right shoulder. The neck was passively correct but associated with severe pain extending her neck. Hoffman’s sign was negative. The cervical radiograph showed severe spondylolisthesis with possible subluxation of C3 on C4 and C4 on C5. CT and MRI were performed to better evaluate the cervical anatomy and deformity, unremarkable for any evidence of fracture, tumor, or instability. The patient decided to pursue conservative therapy such as physical therapy and soft collar.

Case 3: Post-surgical Kyphosis

A 71-year-old female with a past medical history of hypothyroidism was involved in a motor vehicle accident as a restrained driver. The patient was initially evaluated in the emergency department and was allegedly informed that her CT scans of the spine were negative for fracture. She was referred to see us about three months later for neck stiffness and inability to lift her head off her chest. She was neurovascular intact on physical exam. Cervical radiographic imaging, including CT and MRI, showed unstable C4-5 posterior element fracture with ligamentous injury. After an in-depth discussion with the patient about the precarious nature of the injury, we performed C4-5 posterior spinal instrumented fusion (PSIF). Her c-collar was removed two weeks postoperatively. At a six-week follow-up, she presented with chin-on-chest deformity. Her cervical radiographic imaging demonstrated a reduction of the C4-5 ligamentous injury, with global kyphosis from C2 to the upper thoracic spine. There was a concern for hypotonia of the cervical posterior paraspinal musculature. The patient was evaluated by neurology, where she was diagnosed with cervical myotonia likely secondary to injury to the posterior musculature. The patient underwent therapy. At six months post operation, her head position had clinically improved, and she was able to hold her head upright, and no further follow-up was documented.

Case 4: Post-radiation for Lymphoma

The patient was a 66-year-old male with a past medical history significant for radiation for Hodgkin's lymphoma about 40 years ago. He presented with a gradual onset of DHS. On physical exam, he had a flexible deformity of the cervical spine. Cervical and thoracic spinous processes were easily palpable with associated muscle atrophy. A cervical spine MRI demonstrated muscle atrophy with spondylosis. He tried physical therapy. We discussed C2-T5 posterior spinal fusion (PSF) with plastic surgery closure; however, the patient was lost to follow-up.

Case 5: Post-radiation-Induced DHS

The patient was an 80-year-old female with a past medical history significant for radiation treatment to the head and neck for non-Hodgkin's lymphoma about 20 years ago. She presented with 10 years of gradual difficulty with neck extension without pain. On physical exam, she had a passively correctable chin-on-chest deformity. The cervical spine demonstrates prominent spinous processes with muscle atrophy. Cervical X-ray and MRI showed spondylosis. She was not interested in surgical treatment, opted for conservative treatment options with a soft collar, and failed to follow up in the clinic.

Case 6: Neuromuscular DHS

A 74-year-old male with a history significant for Parkinson’s disease, early dementia, and type 2 diabetes presented with an 18-month history of difficulty holding his head up while walking. He tried physical therapy and Botox with minimal relief. Cervical CT showed kyphotic alignment with spondylosis, 39° kyphosis from C2 to C7 on plain films, and 56° kyphosis from C2 to C7 on standing scoliosis films. Physical examination showed the inability to extend the neck to neutral. He was referred to neurology. EMG studies showed motor neuron disease, neuromuscular junction disorder, or generalized myopathy. He was diagnosed with neck flexor dystonia in the setting of his parkinsonism and early dementia. He decided to proceed with PSIF C2-T6. At a four-month follow-up, he was able to hold up his head straighter. The postoperative course was complicated by superficial wound dehiscence and infection requiring operative debridement approximately one year after index surgery. He had recovered well from the washout and maintained strength. He was lost to follow-up and ultimately passed 14 months after the last surgery, and the cause was not well documented.

Case 7: Idiopathic DHS

An 80-year-old male with a past medical history significant for type 2 diabetes mellitus (glycosylated hemoglobin (HbA1c) of 7.0), osteoporosis, and emphysema presented with a case of acute onset of DHS over four weeks. It was associated with pain exacerbated with cervical extension and alleviated with flexion. On physical exam, he had a passively correctable chin-on-chest deformity. The cervical radiograph demonstrated cervical kyphosis centered around a grade 1 spondylolisthesis of C4 on C5 with mild to moderate multilevel degenerative joint disease most outstanding at C5-6. No fractures or lesions were identified on CT. MRI showed no evidence of cervical paraspinal inflammation or fat replacement. No significant laboratory exam findings were present. The patient was referred to neurology, and no neurologic or muscular disease cause was identified for camptocormia. After failed conservative treatment, the patient decided to undergo C3-T3 posterior spinal instrumented fusion with auto iliac crest bone graft at approximately 3.5 months after symptoms onset. At 12 weeks post operation, the patient felt an improved head position. No further follow-up was completed because the patient was diagnosed with prostate cancer at approximately six months post operation and deceased from subsequent postoperative complications from urologic surgery.

Case 8: Post-radiation-Induced DHS

The patient was a 56-year-old male with a past medical history significant for type 2 diabetes mellitus (HbA1c of 7.3) and extensive radiation therapy for Hodgkin's lymphoma, who presented with a multiple-year history of gradual onset of DHS. It was associated with severe midline cervical pain that worsened with prolonged upright posture. He failed extensive physical therapy and bracing support. Physical exam was unremarkable besides a passively correctable chin-on-chest deformity. The cervical radiograph demonstrated mild degenerative changes, with minimal anterolisthesis of C2 on C3 and minimal retrolisthesis of C3 on C4. Cervical CT showed auto fusion from C2 to C3.

Given that the patient failed conservative treatment, we underwent C2-T5 posterior spinal instrumented fusion with auto iliac crest bone graft. His perioperative period was complicated by pressure ulcers surrounding the incision sites, continued neck pain, and difficulty swallowing, especially when extending his neck. Over the next two years, the patient had increased marked proximal kyphosis with a clinical head droop. The patient is currently pending clearance for revision posterior spinal fusion.

Case 9: Post-traumatic DHS

A 78-year-old female with a past medical history of osteoporosis and remote odontoid nonunion fracture presented after a motor vehicle accident with a right C1 ring fracture with rapid progression of DHS while in the hospital. She was discharged from the hospital with a soft cervical collar and home physical therapy. In outpatient follow-up, she complained of severe neck pain with extension and could not wear her collar. On physical exam, she had passively correctable neck kyphosis. A repeat CT scan about one month later showed right C1-C2 facet dislocation, interval subluxation of the odontoid fracture nonunion with severe kyphosis, and chin-on-chest deformity. The patient was brought to the operating room where she was placed in traction; the right C1-2 facet dislocation was reduced, which brought her neck back into normal alignment. She was fixated in this position with a cranial halo vest. The halo vest was removed about two months later, and she was placed in a soft collar. At her two-year follow-up, she complained of neck pain and was referred to cervical rehab. She follows with pain management due to C2 ramus occipital neuralgia and takes buprenorphine patches. She was neurologically intact at that time.

Case 10: Post-traumatic Kyphosis

A 64-year-old male with a past medical history of Parkinson’s disease, mantle cell lymphoma, and metastatic colon cancer presented with progressive kyphosis of his neck after sustaining a fall from bed approximately a month prior. He was experiencing neck pain associated with difficulty swallowing. MRI showed C4 and C5 compression fractures (which were suspected to be pathologic at the time) with stenosis and focal kyphosis. He underwent a C4 and C5 corpectomy and cage placement and C3-6 anterior plating with intraoperative traction, followed by a C2-T1 posterior fusion with C2 and T1 pedicle screws and C3-6 lateral mass screws. Pathology was negative for malignancy and falsely positive for infection (Propionium acnes contaminant). At three-month follow-up, the patient was experiencing worsening neck pain, and CT cervical spine, as well as a Jewett brace and physical therapy, were ordered. Hardware appeared intact on CT. The patient did not feel relief with the neck or Jewett brace and continued to have dysphagia. Dysphagia was likely due to kyphosis and was evaluated by ENT. MRI of the cervical spine was ordered approximately two years postoperatively, but the patient was lost to follow-up.

Case 11: Post-laminoplasty Kyphosis

The patient was a 62-year-old male with a past medical history of prostate cancer who underwent C3-7 decompressive laminoplasty in a different department for spondylotic cervical myelopathy three months prior to presentation in our clinic. He developed a loss of neck mobility and complained of sensations that “something is catching” when he tilted his head. He could not lift his chin to eat or finish a drink and had difficulty swallowing. He was referred to physical therapy for six weeks but instead sought out a second opinion at our clinic. The cervical kyphosis was passively reducible but could not correct past the neutral position on physical exam. Cervical radiography and CT scan demonstrated instability at the C4-5 level with subluxation of the facet joints at C4-5, auto fusion at C3-4, and rigid spine with C5-6 with osteophytic formation (see Supplemental Figure 3). The patient elected to undergo anterior C5-6 corpectomy with structural cage, anterior C4-7 plating, and posterior C2-T1 fusion approximately three months after index surgery as he failed conservative management and had poor subjective quality of life. At a six-month follow-up, he was walking normally with no complaints of myelopathy and minimal neck pain. At eight years follow-up, he remained clinically stable without deterioration.

Case 12: Post-surgical DHS

A 79-year-old male with a past medical history significant for borderline diabetes presented to the emergency department with neck pain after losing his balance and hitting his head on concrete. Cervical spine CT showed a type II odontoid fracture and a type III Jefferson fracture. He was treated in a Miami J collar. At a four-month follow-up, the patient had nonunion of both fractures. A decision was made to pursue C1-C2 fusion given that the patient had frequent falls, which placed him at high risk of significant spinal cord injury living with the nonunion fracture. At five-month postoperative follow-up, the patient had substantial cervical kyphosis with almost a chin-on-chest deformity. On physical exam, the neck was passively correctable, and we were able to actively extend the neck to neutral. No EMG study was done. He was diagnosed with severe global cervical kyphosis secondary to cervical myotonia occurring after C1-C2 posterior fusion surgery for odontoid fracture, with kyphosis attributed to myotonia. Eight months after the original surgery, the patient underwent removal of hardware, followed by occipital to T3 posterior instrumented fusion (Supplemental Figure 4). At six months post operation, the patient had improvement in his symptoms and acceptable clinical kyphosis. At a three-year follow-up, the patient complained of shuffling gate and axial neck pain. He was referred to physical therapy for balance training and muscle strengthening.

Case 13: Post-surgical DHS

A 63-year-old female with a past medical history of post-polio syndrome and past surgical history of C3-C4 anterior cervical discectomy and fusion (ACDF) presented to the emergency department for severe multilevel cervical stenosis, subluxations, and evidence of progressive cervical myelopathy in the setting of rheumatoid disease. She was treated with a C3-C7 laminectomy, and C2-T1 PSIF for cervical degenerative spinal stenosis. At a two-month follow-up, one of the C2 screws showed uncoupling on X-ray imaging. She was diagnosed with post-laminectomy kyphosis. At four months post operation, she underwent the removal of T1 and C2 hardware with an extension of fusion from C2 to T5 for cervicothoracic pseudoarthrosis with hardware failure. She continued to do well from the cervicothoracic perspective but developed leg weakness secondary to lumbar stenosis. She was lost to follow-up approximately one year postoperatively.
